# Individual and household factors associated with incidences of village malaria in Xepon district, Savannakhet province, Lao PDR

**DOI:** 10.1186/s41182-017-0077-2

**Published:** 2017-11-07

**Authors:** Nouhak Inthavong, Daisuke Nonaka, Sengchanh Kounnavong, Moritoshi Iwagami, Souraxay Phommala, Jun Kobayashi, Bouasy Hongvanthong, Tiengkham Pongvongsa, Paul T. Brey, Shigeyuki Kano

**Affiliations:** 1 Department of Global Health, Graduate School of Health Sciences, University of the Ryukyus, Uehara 207, Nishihara-cho, Okinawa, 903-0215 Japan; 2 SATREPS Project for Parasitic Diseases, Vientiane, Lao People’s Democratic Republic; 3 National Institute of Public Health, Ministry of Health, Ban Kaognot, Samsenthai Road, Sisattanak District, Vientiane, Lao People’s Democratic Republic; 4 Department of Tropical Medicine and Malaria, Research Institute, National Center for Global Health and Medicine, 1-21-1 Toyama, Shinjuku-ku, Tokyo, 162-8655 Japan; 5 Institut Pasteur du Laos, Ministry of Health, Sisattanak District, Vientiane, Lao People’s Democratic Republic; 6 Center of Malariology, Parasitology and Entomology, Ministry of Health, Vientiane, Lao People’s Democratic Republic; 7 Savannakhet Provincial Health Department, Thahea village, Kaysone-Phomvihan District, Savannakhet, Lao People’s Democratic Republic

**Keywords:** Malaria, Incidence, Risk factor, Behavior and Laos

## Abstract

**Background:**

In the Lao PDR, the incidence of malaria greatly differs among villages even within a subdistrict, and the reasons for this difference are poorly understood. The objective of this study was to identify differences in villagers’ behavior and the household environment between villages with high incidences and those with low incidences of malaria in a rural district of the Lao PDR.

**Methods:**

A case-control study was conducted in Xepon district, Savannakhet province. Case villages were defined as those with a high incidence (> 10 cases per 1000 population per year), and control villages were those with a low incidence (0–10 cases per 1000 population per year). Data were collected from 178 households in the six case villages and six control villages between December 2016 and January 2017. The data collection consisted of an interview survey with the heads of households and an observational survey in and around the house. Logistic regression was used to assess the association between the case-control status and individual-level behavioral factors and household-level environmental factors adjusted for socio-demographic and economic factors.

**Results:**

Compared to the household members in the control villages, household members in the case villages were significantly more likely to work at night in the forest (adjusted odds ratio 1.95; 95% confidence interval 1.28 to 2.98) and more likely to sleep overnight in the forest (adjusted odds ratio 1.94; 95% confidence interval 1.13 to 3.33). Additionally, compared to the households in the control villages, households in the case villages were significantly more likely to have an open space on the house surface (adjusted odds ratio 3.64; 95% confidence interval 1.68 to 7.84).

**Conclusions:**

There were significant differences in nighttime working and sleeping behaviors in the forest and the presence of an open space on the house surface in the case versus control villages. These differences can partly explain the difference in the incidences of malaria among the villages. The Lao National Malaria Control Program should recommend that villagers use personal protection when working and sleeping in the forest and to reduce any open space on the house surfaces.

## Background

**Fig. 1:**
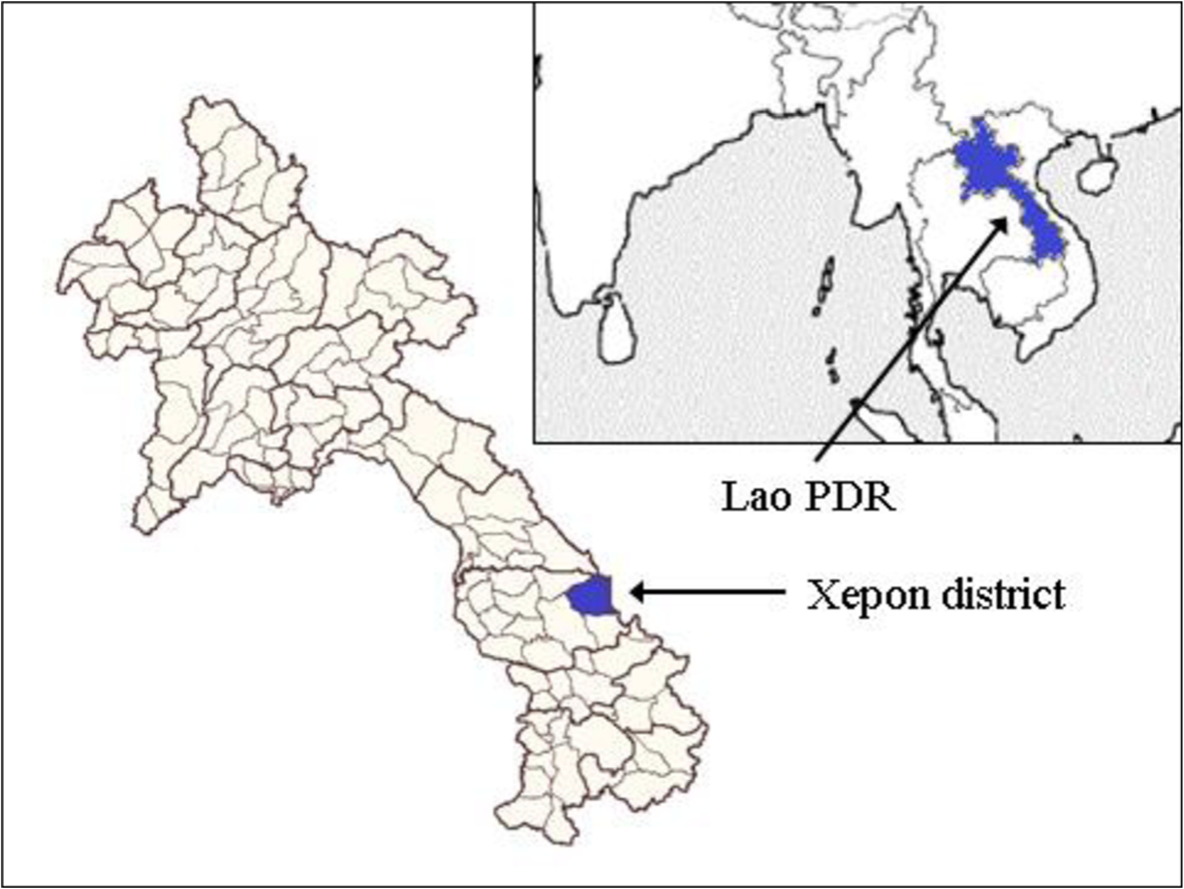
The location of the Lao PDR and Xepon district

The Lao People’s Democratic Republic (Lao PDR) is a lower-middle-income country in Southeast Asia, bordering with China on its north border, Vietnam in the east and northeast, Cambodia in the south, Thailand in the west, and Myanmar in the northwest (Fig. 1). The country comprises 18 provinces with 148 districts and 8500 villages. The total land area is 23.68 million hectares, 79% of which is mountainous. The population of the Lao PDR was 7.0 million in 2016 [1], of which 80% live in rural areas and 85–90% are dependent upon subsistence farming [2].

Since 1992, the Lao PDR has implemented a nationwide malaria control program. The current malaria control strategies emphasize the promotion of long-lasting insecticide-treated bed nets (LLINs), early diagnosis by microscopic examination and rapid diagnostic tests, and prompt treatment with an artemisinin-based combination therapy (ACT). Since 2008, the use of the ACT has gradually been scaled-up to cover the entire public health sector, including village health volunteers (community health workers), some businesses in the private sector, and registered private pharmacies [3].

Since 2010, the Lao National Malaria Control Program has adopted stratification-based planning and implementation of control activities; villages are stratified into three strata according to village-level incidences of malaria, and different control strategies are applied to different strata. In stratum I villages, which are defined by an annual incidence of less than 0.1 cases per 1000 population, control activities focus on the maintenance of existing bed nets. In stratum II villages, which are defined by an annual incidence of 0.1 to 10 cases per 1000 population, control activities include the distribution of LLINs and the implementation of village-level diagnosis and treatment with rapid diagnostic tests and ACTs, respectively, and the maintenance of existing bed nets. In stratum III villages, which are defined by an annual incidence of more than 10 cases per 1000 population, additional control activities are the distribution of single LLINs to mobile members of the population and the provision of insect repellent [3].

During the period between 2000 and 2010, the Lao PDR significantly reduced its malaria burden by reducing the number of annual malaria deaths from 350 to 24. The number of confirmed cases had also declined from around 75,000 cases in 2000 to 30,000 cases in 2010. However, the national annual parasite incidence rose from 2.66/1000 population in 2011 to 7.3/1000 population in 2014 [4]. Currently, the malaria burden is concentrated in the southern provinces; approximately 90% of the malaria cases were reported in the five southernmost provinces, i.e., Savannakhet, Saravan, Sekong, Attapeu, and Champasak [3, 5].

In a total of 15 districts in Savannakhet province, malaria was endemic in four districts including Xepon district, which is located on the Vietnamese border, approximately 600 km to the southeast of the Vientiane capital. Xepon has a total area of 21,774 km^2^ and 45,000 people. According to the Xepon District Health Office, most of them are ethnic minorities, specifically the Tri and Mangkong people, comprising 75% of the total district population. They have their own distinctive languages, have limited formal education, and live in mountainous and forested areas that are far from health care facilities. The majority of the population is farmers engaged in rice farming. Their houses are surrounded by vegetation and puddles, and cattle are often kept in fenced enclosures near the houses. According to the surveillance data obtained from the Center of Malariology, Parasitology and Entomology, the Xepon District Hospital recorded 225 cases of malaria including 208 *Plasmodium falciparum* mono-infections, 16 *Plasmodium vivax* mono-infections, and 6 co-infections of *P. falciparum* and *P. vivax* in 2015.

A number of studies conducted in the Lao PDR or neighboring countries have emphasized the importance of behavioral and environmental factors on the risk of malaria infection. In the Lao PDR, inappropriate use of bed nets [6] and sleeping away from home (e.g., sleeping in a farming hut) have been reported as risk factors [7, 8]. In Thailand, the relative risk of malaria infection was three times higher among people who slept in farm huts than in people staying in residential villages [9]. In Sri Lanka, the incidence of malaria among residents of poor-quality housing was higher compared with a population living in improved housing [10]. A study conducted in the Lao PDR also showed that house construction material, veranda style, kitchen location, and cow ownership were significantly associated with the house entry of *Anopheles* mosquitoes [11]. In addition, a study in Vietnam mentioned that living in a wooden/bamboo house was significantly related to malaria infection [12]. Hence, the present study hypothesized that individual behavioral factors and household environmental factors would be significantly different between villages with high incidences and villages with low incidences of malaria in Xepon district, Savannakhet province, Lao PDR.

## Methods

### Study site and population

**Fig. 2:**
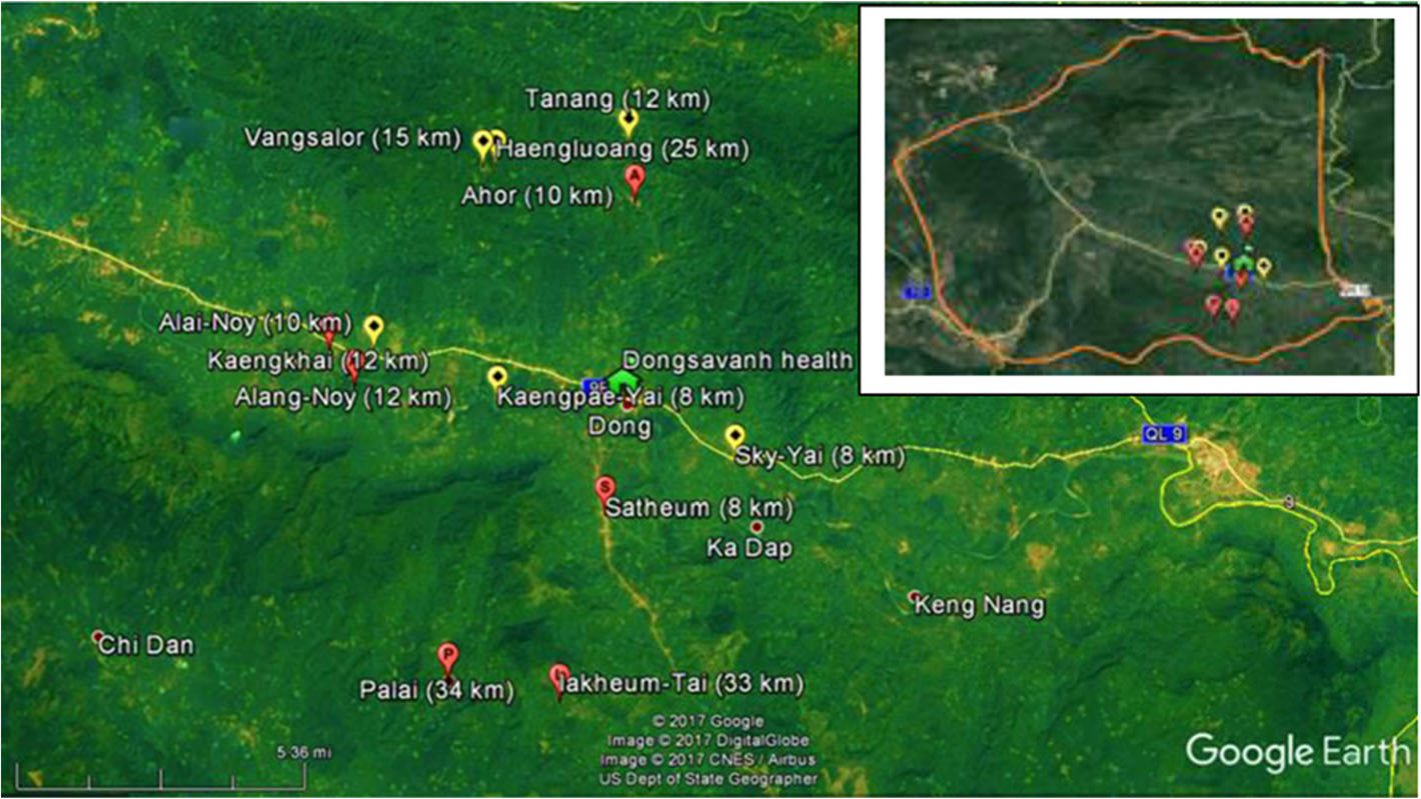
Map of the study villages. The maps show the villages in which the present study was conducted. The red points indicate the locations of case villages, and the yellow points indicate the locations of control villages along with the distance of each from Xepon district hospital according to the hospital’s data

This was a case-control study that was conducted in the 12 villages in the catchment area of the Dongsavanh Health Center, Xepon district (Fig. 2). A case village was defined as a stratum III village, and the control villages were defined as stratum I or II villages. Of the 43 villages in the catchment area, 13 stratum I and II villages were excluded, either because the number of households was less than the required number for a survey or because these villages were located near the health center and were thus not comparable to stratum III villages that were located far from the health center. Then, 6 case villages were randomly selected from 19 stratum III villages. Similarly, 6 control villages were randomly selected from 11 stratum I and II villages, 3 from the 7 stratum I villages and 3 from the 4 stratum II villages. The number of villages and households to be selected was mainly determined by the availability of human and time resources.

In each of the selected villages, 15 households were randomly selected, using a household list obtained from the heads of the villages; however, the survey failed to include 15 households in one village. All members of the selected households were included. Overall, this study collected data on 1070 individuals from 178 households.

The names (and number of eligible households) of the selected case villages were Lakheum-Tai (26), Palai (52), Alang-Noy (22), Satheum (53), Ahor (39), and Kaengkhai (43), and those of the selected control villages were Haengluoang (17), Kaengpae-Yai (18), Sky-Yai (60), Vangsalor (20), Tanang (33), and Alai-Noy (34).

### Data collection

Data were collected between December 2016 and January 2017 through an interview survey and an observational survey. The trained surveyors, who were health workers at the Xepon District Health Office, visited the selected households and conducted interviews with the heads of households. Information on every member of each household was collected via the heads of households. When the head of a household was absent during the survey, another adult member of the household (e.g., spouse of the head) was recruited. When the respondent was unable to communicate with surveyors in the Lao language, the head of the village or other villagers who were able to speak both the local language and the Lao language were asked to assist the surveyors. For the observational survey, surveyors observed the household characteristics in and around the houses using a checklist.

### Factors and measurements

**Fig. 3:**
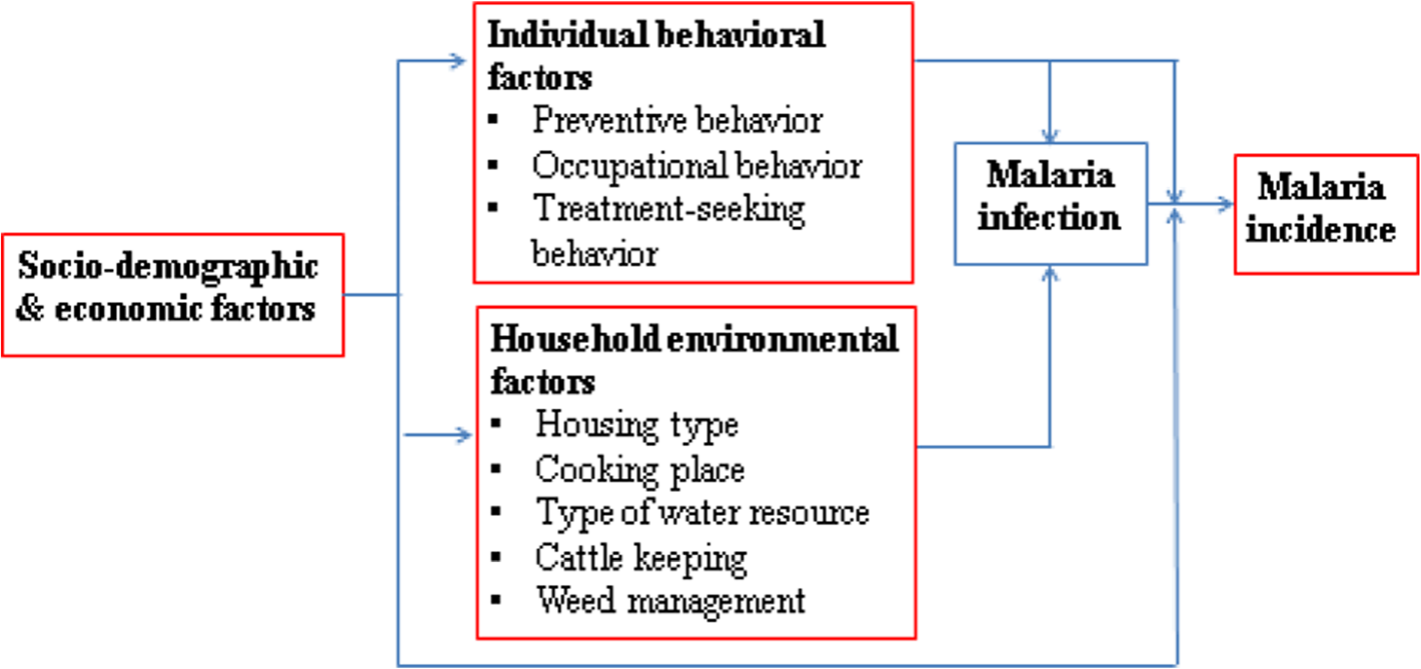
Conceptual framework

Selection of the factors that were measured in this study was based on the conceptual framework depicted in Fig. 3. The conceptual model was developed principally on the basis of the findings of malaria studies that were conducted in the Lao PDR or neighboring countries [6–14]. The interview survey measured socio-demographic and economic factors and individual behavioral factors, whereas the observational survey measured household environmental factors.

Individual behavioral factors included preventive behavior (i.e., frequency of sleeping under a bed net in the past month), occupational behavior (i.e., experience of working in the forest at night or early morning in the past 1 year, sleeping in the forest in the past 1 year), and seeking behavior for malaria treatment in the past 1 year.

Household environmental factors included housing structure (i.e., wall and floor materials); presence of an open space that was larger than the size of an A4 sheet of paper on the surface of the house (i.e., lack of a door or no window coverings, no ceiling, and open eaves); kitchen location; type of water resources for bathing, washing clothes, and other daily use; keeping cattle near the house; presence of weeds within 10 m of the house in the household compound; and household bed net density (i.e., person per net ratio defined as the number of household members divided by the number of available bed nets in the household [15]). When measuring the presence of an open space, surveyors paid attention to and included only an open space that was larger than approximately the size of an A4 sheet of paper, ignoring an open space that was smaller than approximately the size of an A4 sheet of paper.

Socio-demographic factors included age, gender, and number of members in each household. Socio-economic factors included educational attainment, occupation of each member of the households, and household relative wealth determined by the household possession of assets including a motorbike, mobile phone, television, DVD/CD, car/tractor, and refrigerator. Principal component analysis was used to assess the weight of household assets by scoring factors for wealth index variables (Table 1) and to build a household wealth index [16]. Households were ranked by the household wealth index and divided into quartiles. As a result of the analysis, six components were extracted and the first component, which explained 41.3% of the total variance with the eigenvalue of 2.48, was used for the wealth index. For each asset variable, a scoring factor was derived from the weight of a variable divided by the eigenvalue. The wealth index of a household was expressed as the sum of the product between the scoring factor of an asset variable and the household’s standardized score of the variable.

**Table 1:** Scoring factors for wealth index variables

Variables	Scoring factor
Car/tractor	0.216
Motorbike	0.232
Mobile phone	0.262
Television	0.323
DVD/CD	0.301
Refrigerator	0.198

### Data analysis

Frequencies, percentages, and medians with interquartile ranges were computed to summarize the data. Bivariate analyses were conducted to assess an association between a predictive factor and the case-control status, using Fisher’s exact test. Multivariate analyses that adjusted for the effect of other predictive factors were conducted to assess an association between a predictive factor and the case-control status using logistic regression. According to Katz [17], regardless of a *p* value obtained from the bivariate analyses, variables that are theoretically important or those that have been reported as risk factors were included in the multivariate model. Two multivariate models were built: one included individual-level predictive variables and the other included only household-level predictive variables. The amount of multicollinearity (i.e., two or more predictive variables are correlated each other) in the multivariate model was estimated by the variance inflation factor (VIF). A VIF ≥ 4 was considered as the sign of multicollinearity. A *p* value < 0.05 was considered statistically significant. Statistical analyses were performed using SPSS version 24.

## Results

### Associations between individual-level characteristics and case-control status

The proportion of household members who worked in the forest at night was significantly higher in the case villages than in the control villages (21.8 vs. 15.6%, *p* = 0.010) (Table 2). In contrast, the proportion of household members who worked in the forest in the early morning was significantly lower in the case villages than in the control villages (19.1 vs. 24.4%, *p* = 0.038). Additionally, the proportion of household members who slept overnight in the forest (12.4 vs. 8.1%, *p* = 0.026), who had a malaria episode in past 1 year (13.8 vs. 6.3%, *p* = <0.001), and who sought a place for malaria treatment (48.7 vs. 0.0%, *p* = <0.001) was significantly higher in the case villages versus the control villages. No significant differences were observed in gender, age, educational attainment, occupation, and frequency of using a bed net.

**Table 2:** Bivariate analysis of differences in individual-level characteristics

Characteristics	Respondents in cases (*n* = 550)		Respondents in controls (*n* = 520)		*p* value^a^
	*n*	%	*n*	%	
Gender					
Male	272	49.5	261	50.2	0.854
Female	278	50.5	259	49.8	
Age					
< 15 years	271	50.4	239	47.0	0.293
≥ 15 years	267	49.6	270	53.0	
Educational attainment					
No formal education	389	70.7	367	70.6	1.000
Primary school or higher	161	29.3	153	29.4	
Occupation					
Farmer	290	52.7	275	52.9	0.978
Child	159	28.9	152	29.2	
Student	101	18.4	93	17.9	
Frequency of using a bed net					
Everyday	455	82.7	428	82.3	0.872
Sometimes/never	95	17.3	92	17.7	
Working in the forest at nighttime in the past year					
No	430	78.2	439	84.4	0.010
Yes	120	21.8	81	15.6	
Working in the forest in the early morning in the past year					
No	445	80.9	393	75.6	0.038
Yes	105	19.1	127	24.4	
Sleeping overnight in the forest in the past year					
No	482	87.6	478	91.9	0.026
Yes	68	12.4	42	8.1	
Malaria episode in past 1 year					
No	474	86.2	487	93.7	< 0.001
Yes	76	13.8	33	6.3	
Place for the treatment of the episode					
Health center	36	47.4	30	90.9	< 0.001
Village health volunteer	37	48.7	0	0.0	
Other	3	3.9	3	9.1	

^a^Fisher’s exact test

Among the household members who worked in the forest at night, the proportion of household members who slept overnight in the forest was not significantly different between the case and control villages (43.3 vs. 48.1%, *p* = 0.564) (data not shown in table).

After adjusting for the effects of other individual-level predictive variables in the multivariate analysis, the associations observed in the bivariate analysis remained significant (Table 3); working in the forest at night (adjusted odds ratio [AOR] 1.95; 95% confidence interval [CI] 1.28 to 2.98), working in the forest in the early morning (AOR 0.51; 95% CI 0.36 to 0.73), and sleeping overnight in the forest (AOR 1.94; 95% CI 1.13 to 3.33). Although the difference in age groups was not significant in the bivariate analysis, it became significant in the multivariate analysis (AOR 0.61; 95% CI 0.38 to 0.98). There was no sign of multicollinearity between the variables in the multivariate analysis.

**Table 3:** Multivariate analysis of differences in individual-level characteristics and case-control status

Predictive factors	Crude odds ratio (95% CI)	Adjusted odds ratio^a^ (95% CI)
Gender		
Male	1.00	1.00
Female	0.97 (0.76–1.23)	1.22 (0.93–1.61)
Age		
< 15 years	1.00	1.00
≥ 15 years	0.87 (0.68–1.11)	0.61 (0.38–0.98)
Education attainment		
No formal education	1.00	1.00
Primary school and higher	0.99 (0.76–1.29)	0.88 (0.58–1.32)
Occupation		
Child	1.00	1.00
Student	1.03 (0.72–1.48)	1.33 (0.78–2.25)
Farmer	1.00 (0.76–1.33)	1.46 (0.87–2.45)
Frequency of using bed net		
Everyday	1.00	1.00
Sometimes/never	0.97 (0.70–1.33)	0.80 (0.57–1.14)
Working in the forest at nighttime in the past year		
No	1.00	1.00
Yes	1.51 (1.10–2.06)	1.95 (1.28–2.98)
Working in the forest in the early morning in the past year		
No	1.00	1.00
Yes	0.73 (0.54–0.97)	0.51 (0.36–0.73)
Sleeping overnight in the forest in the past year		
No	1.00	1.00
Yes	1.60 (1.07–2.40)	1.94 (1.13–3.33)

^a^Adjusted for age, gender, occupational attainment, education, frequency of using a bed net, working in the forest at nighttime in the past year, working in the forest in the early morning in the past year, and sleeping overnight in the forest in the past year

### Associations between household-level characteristics and case-control status

The proportion of households that used flooring materials other than wood was significantly lower in the case villages than in the control villages (21.1 vs. 36.4%, *p* = 0.031). The proportion of households with an open space was significantly higher in the case villages than in the control villages (77.8 vs. 50.0%, *p =* <0.001) (Table 4). No other significant differences were found in the bivariate analysis.

**Table 4:** Bivariate analysis of the associations between household-level characteristics

Characteristics	Respondents in cases (*n* = 90)		Respondents in controls (*n* = 88)		*p* value^a^
	*n*	%	*n*	%	
Number of household members					
< 5	27	30.0	27	30.7	0.994
5–7	40	44.4	39	4.3	
≥ 8	23	25.6	22	25.0	
Median (interquartile range)	6	4–8	6	4–8	
Household that hung a bed net					
No	0	0.0	4	4.5	0.058
Yes	90	100	84	95.5	
Household that hung long-lasting insecticide-treated bed net					
No	3	3.3	9	10.2	0.079
Yes	87	96.7	79	89.8	
Person per net ratio					
≤ 2.5	49	54.4	44	52.4	0.879
> 2.5	41	45.6	40	47.6	
Type of water used for daily life					
Stream water	41	45.6	44	50.0	0.653
Water other than stream water	49	54.4	44	50.0	
Wealth index quartiles					
First (least poor)	19	21.1	30	34.1	0.121
Second	21	23.3	22	25.0	
Third	18	20.0	17	19.3	
Fourth (poorest)	32	35.6	19	21.6	
Wall materials					
Brick	48	53.3	45	51.1	0.881
Other	42	46.7	43	48.9	
Flooring materials					
Wood	71	78.8	56	63.6	0.031
Other	19	21.1	32	36.4	
Roofing materials					
Zinc	82	91.1	73	83.0	0.121
Other	8	8.9	15	17.0	
Open space in the house (size larger than A4 sheet)					
No	20	22.2	44	50.0	< 0.001
Yes	70	77.8	44	50.0	
Percent of open spaces in the house (%)					
≤ 10	23	32.9	13	29.5	0.837
> 10	47	67.1	31	70.5	
Median (interquartile range)	11.2	0.9–21.5	0.6	0.0–18.4	
Kitchen location					
Inside the house	58	64.4	61	69.3	0.527
Outside the house	32	35.6	27	30.7	
Cattle keeping near the house					
No	53	58.9	58	65.9	0.357
Yes	37	41.1	30	34.1	
Weed management within 10 m around the house					
Poor	52	57.8	42	47.7	0.230
Good	38	42.2	46	52.3	

^a^Fisher’s exact test

After adjusting for the effects of other household-level variables in the multivariate analysis, the association with flooring materials other than wood was significant (AOR 0.25; 95% CI 0.09–0.68) (Table 5). The presence of an open space also remained significant (AOR 3.64; 95% CI 1.68 to 7.84). Although the bivariate analysis did not show a significant difference in the proportion of household wealth quartiles, the multivariate analysis showed that compared to the proportion of the first-quartile households, the proportion of the fourth-quartile households was significantly higher in the case villages than in the control villages (AOR 6.16; 95% CI 1.92 to 19.72). There was no sign of multicollinearity between the variables in the multivariate analysis.

**Table 5:** Multivariate analysis of the associations between household characteristics and case-control status

Predictive factors	Crude odds ratio (95% CI)	Adjusted odds^a^ ratio (95% CI)
Number of household members		
≤ 6	1.00	1.00
> 6	1.16 (0.63–2.12)	1.48 (0.70–3.13)
Person per net ratio		
≤ 2.5	1.00	1.00
> 2.5	0.92 (0.50–1.67)	0.83 (0.40–1.71)
Type of water used for daily life		
Stream water	1.00	1.00
Water other than stream water	1.19 (0.66–2.15)	1.50 (0.70–3.21)
Wealth index quartiles		
First (least poor)	1.00	1.00
Second	1.50 (0.65–3.45)	1.67 (0.63–4.38)
Third	1.67 (0.69–4.02)	2.35 (0.77–7.14)
Fourth (poorest)	2.65 (1.18–5.96)	6.16 (1.92–19.72)
Wall materials		
Brick	1.00	1.00
Other	0.96 (0.50–1.64)	0.97 (0.40–2.35)
Flooring materials		
Wood	1.00	1.00
Other	0.46 (0.24–0.91)	0.25 (0.09–0.68)
Roofing materials		
Zinc	1.00	1.00
Other	0.47 (0.19–1.18)	0.61 (0.21–1.75)
Open space in the house		
No	1.00	1.00
Yes	3.50 (1.82–6.70)	3.64 (1.68–7.84)
Kitchen location		
Inside the house	1.00	1.00
Outside the house	1.24 (0.66–2.33)	0.94 (0.35–2.48)
Cattle keeping near the house		
No	1.00	1.00
Yes	1.35 (0.73–2.48)	1.78 (0.85–3.74)
Weed management within 10 m around the house		
Poor	1.00	1.00
Good	0.66 (0.36–1.20)	0.67 (0.32–1.39)

^a^Adjusted for number of household members, person per net ratio, type of water used for daily life, wealth index, wall materials, flooring materials, roofing materials, open space in the house, kitchen location, cattle keeping near the house, weed management within 10 m around the house

### Risk behaviors in households

There was no tendency that risk behaviors concentrated in some particular households; 68.9% (62/90) of the households in the case villages and 67.0% (59/88) of the households in the control villages had at least one member who worked in the forest at night. Additionally, 51.1% (46/90) of the households in the case villages and 37.5% (33/88) of the households in the control villages had at least one member who slept in the forest at night (data not shown in table).

## Discussion

This study found that a number of individual-level and household-level factors were associated with village-level incidences of malaria. The results showed that the household members in the case villages were significantly more likely to work at night and more likely to sleep overnight in the forest, as compared with those in the control villages. These differences of behaviors could be the reason why the incidences of malaria were higher in the case villages than in the control villages. A case-control study conducted in Attapeu province, Lao PDR reported that study participants who slept away from home in the past 2 weeks had significantly higher odds of malaria infection than those who did not [7, 8]. A cross-sectional study that was conducted in Savannakhet province, Lao PDR reported that staying overnight in the forest was associated with *P. viviax* mono-infections [18]. A cross-sectional study conducted in Vietnam reported that working and sleeping at night in the forest was a risk factor for malaria infection [13], and a similar finding was also reported from Thailand [14]. In the study district, *Anopheles dirus* is thought to be the main malaria vector [19]. The biting time of *A. dirus* is between 19:00 and 06:00 with the peak at midnight [8, 20]. This vector behavior can also support our causal inference that the statistical associations with working and sleeping at night in the forest represent a cause-effect relation.

In rural areas of the Lao PDR, people work in the forest during nighttime to sustain their lives. The purposes of nighttime working in the forest include seeking forest products, hunting, and protecting crops in a slash-and-burn field from animals. Therefore, it is difficult to stop their behavior. Considering the risk of malaria infection of the behavior, the Lao National Malaria Control Program has already promoted devices for protection. Mosquito repellent lotions and single LLINs for this mobile population have been made available in the stratum III villages where the incidences of malaria are high. However, little is known about whether the villagers are actually using these devices. In addition to these protective devices, wearing long-sleeved shirts should be recommended to villagers who work in the forest at nighttime. A cross-sectional study that was conducted with hill tribes in Thailand showed that when working outdoors, people who wore long-sleeved clothes were significantly less likely to have malaria infection, as compared with those who did not [21].

The present study also showed that the houses in the case villages were significantly more likely to have an unprotected open space on their surfaces, as compared to the houses in the control villages. The difference in the presence of an open space between the case and control houses could be one reason for the higher incidence of malaria in the case villages than in the control villages. Such open spaces can make it easier for vector mosquitoes to enter the house, thus increasing human-vector contacts. Although no study has been conducted in the Lao PDR and neighboring countries, a number of studies conducted in Sri Lanka or African countries have shown an association between the presence of an open space or non-screened space on the surface of a house and malaria infections among the residents of the house [10, 22–25]. An open space under the eaves is considered to be the most critical for house entry of malaria vectors [23, 25, 26]. A randomized controlled trial of house screening intervention showed that the number of mosquitoes inside the house and the incidences of childhood anemia were significantly lowered in the intervention group [23].

There are at least two concerns about promoting net screens in the study district. One is the increased temperature due to installing net screens. A study in Gambia showed that houses with net screens were a little hotter than houses without any screens; full screened houses were 0.26 °C hotter at night than houses with screened ceilings and 0.51 °C hotter than houses with no screening (28.43 °C). Nonetheless, only 9% of full screened house users complained about the heat [27]. Another concern is the cost for installing net screens. Because many households in malaria-prone villages of the study district may not afford to buy nets for screening by themselves, the Lao National Malaria Control Program should consider subsidizing a part of the cost. The Lao National Malaria Control Program has already experienced the distribution of bed nets and repellent spays to the target population at a subsidized price.

Although the difference in household bed net density measured by person per net ratio was not significant between the case and controls, it should be noted that 45.6% of the households in the case villages did not possess an adequate number of bed nets to protect all members of the household. This finding suggests the need for increasing the availability of LLINs in malaria-prone villages.

In the present study, the poorest households were significantly more abundant in the case villages than in the control villages. It has been frequently reported from many countries that poorer households are at higher risk of malaria [26, 28, 29]. In this study site, there are at least two possible explanations for why poor households are at risk of malaria. First, poor households would be more reluctant to seek treatment for malaria as compared to non-poor households. Although treatment for malaria is free of charge in the Lao PDR, treatment seeking requires opportunity costs including transportation costs. Second, as compared to better-off households, poor households would be more likely to be exposed to malaria infection because protective materials including long-sleeved clothes or insecticides are less likely available among these households.

The present study compared individual and household characteristics between the case villages and control villages. The result showed that there were significant differences in these characteristics. However, because the case villages and control villages were not necessarily comparable, caution is necessary when interpreting the results. If case and control individuals are recruited in the same village, then there might be no significant differences in individual and household characteristics. However, a previous case-control study that recruited cases and controls in the same village in Lao PDR also showed that there were significant differences in individual and household characteristics, i.e., not using a bed net, sleeping away from home, and houses closed to a vector breeding site [8].

A cross-sectional study that was conducted in Xepon district by Pongvongsa et al. showed that malaria infections were highly clustered at the household level. Pongvongsa et al. suggested two possible reasons for such household clustering. One is that household members share the same environmental risk factors including the proximity of housing location to breeding sites and housing type. Another is that household members share the same risk behaviors such as not using a bed net and working in the forest [30]. The results of the present study partly support their speculation. The present study found that houses with an open space were significantly more common in villages with high incidences than in those with low incidences of malaria, suggesting the importance of housing conditions. The present study also found that some households did not own any LLINs, suggesting that all members of the households are equally not protected by nets. The present study, however, did not find that risk behaviors were highly clustered at the household level; individuals who practiced risk behaviors did not concentrate on particular households.

There are four limitations in the present study. Firstly, the case villages were not matched with the control villages on the basis of village-level characteristics. Therefore, the risk of malaria infection could be different between the two. The stratum III case villages were more likely to be located far from the health center as compared with the stratum I and II control villages. Thus, the case villages were likely to be at higher risk of malaria infections, as compared to control villages. The ethnicity of the study population was, however, the same between the case and control villages, i.e., Tri and Mangkong. To minimize the impact of the absence of matching, the present study excluded the stratum I and II villages that were located within 7 km of the health center. Secondly, although people in the study site might have sought malaria treatment from the private sector, the village-level incidence of malaria used in the present study relied solely on records from the public health sector. Thus, the present study could underestimate the village-level incidence. However, the impact of the underestimation would be very low because access to the private health sector was available only in the center of Xepon district, which was located at least 8 km from the study villages, and as shown in Table 2, more than 90% of the study households reported treatment seeking from the public sector for past malaria episodes. Thirdly, because in the present study the outcome measurement was not at the individual level but at the village level, the present study was unable to take the village and household clustering into account in the analyses. Therefore, the present study possibly overestimated the significance of the statistical associations. Finally, because small villages in which the total number of households was 14 or fewer were excluded, the results of the present study were not free from selection bias. However, the impact of the selection bias would be small as the total number of households was 15 or larger in the most of the villages of the study district.

Although in the present study the study site was confined to the catchment area of one health center in Xepon district, the findings of the present study can be generalized to a wider area in Savannakhet province. This is because the same ethnic groups such as Tri and Mangkong are predominant in the malaria-prone villages of the province including the study villages of the present study. Additionally, ecological characteristics are similar among the malaria-prone villages of the province.

## Conclusion

There were significant differences in personal behaviors and household environment between the villages with high and those with low incidences of malaria. Villagers in the villages with high incidences of malaria were significantly more likely to work and sleep in the forest at night as compared with those in villages with low incidences. Houses in villages with high incidences were significantly more likely to have open spaces on their surfaces that could facilitate mosquito entry. These differences can partly explain the difference in the incidences of malaria between the villages. The Lao National Malaria Control Program should recommend that villagers use personal protection such as repellent lotions, single LLINs, and long-sleeved clothes when they work and sleep in the forest and to reduce open spaces on house surfaces by installing net screens.
